# Oral–Systemic Health Awareness Among Physicians and Dentists in Croatian Primary Healthcare: A Cross-Sectional Study

**DOI:** 10.3390/epidemiologia6030043

**Published:** 2025-08-07

**Authors:** Marija Badrov, Martin Miskovic, Ana Glavina, Antonija Tadin

**Affiliations:** 1 Department of Restorative Dental Medicine and Endodontics, Study of Dental Medicine, School of Medicine, University of Split, 21000 Split, Croatia; marija.badrov@mefst.hr (M.B.); mm91925@mefst.hr (M.M.); 2 Department of Oral Medicine, Study of Dental Medicine, School of Medicine, University of Split, 2100 Split, Croatia; glavina2014@gmail.com; 3 Department of Dental Medicine, Department of Maxillofacial Surgery, University Hospital of Split, Spinciceva 1, 21000 Split, Croatia

**Keywords:** dental practitioner, knowledge, medical doctor, oral health, oral manifestations, periodontal disease, systemic health

## Abstract

Objectives: This study aimed to assess the knowledge, attitudes, and self-confidence of physicians and dentists in Croatia regarding the relationship between oral and systemic health, focusing on periodontal disease and oral manifestations of systemic diseases. Methods: A cross-sectional, web-based survey was conducted among physicians and dentists in Croatian primary healthcare. The questionnaire addressed six thematic domains, including demographic information, knowledge, self-assessment, and clinical practice. Descriptive and comparative statistical analyses were performed. Results: A total of 529 respondents were included (291 physicians and 238 dentists). The mean knowledge score for the association between periodontitis and systemic diseases was 6.8 ± 3.6 out of 15, indicating limited knowledge. For oral manifestations of systemic diseases, the mean score was 10.0 ± 3.8 out of 16, reflecting moderate proficiency. Dentists scored higher than physicians in both domains, though not significantly (*p* > 0.05). Routine oral mucosal examinations were reported by 89.5% of dentists and 43.0% of physicians (*p* ≤ 0.001). Only 21.3% of physicians correctly identified the link between periodontitis and adverse pregnancy outcomes, compared to 58.8% of dentists. The primary barriers to effective clinical management were a lack of experience (52.7%) and inadequate education. While 68.3% of dentists felt adequately educated on oral–systemic links, only 22.7% of physicians reported the same. Conclusions: Significant gaps in knowledge and confidence were observed, particularly among physicians. These findings underscore the need to integrate oral–systemic health topics into medical education and to promote interprofessional collaboration to improve patient outcomes.

## 1. Introduction

Oral diseases impact systemic health through both biological and non-biological mechanisms. The oral microbiome, along with its metabolic by-products, can translocate into the bloodstream, triggering systemic inflammation and modulating immune responses. Among oral diseases, periodontitis is most prominently associated with systemic conditions, primarily via bacteremia and persistent chronic inflammation [1,2,3]. This association is further influenced by factors such socioeconomic status, health literacy, access to dental care, oral hygiene practice, lifestyle behaviors, and psychosocial stressors. Periodontal diseases, including gingivitis and periodontitis, are highly prevalent and have been implicated in the onset or progression of various systemic conditions, including diabetes, cardiovascular disease, metabolic syndrome, Alzheimer’s disease, rheumatoid arthritis, adverse pregnancy outcomes, respiratory infections, and certain malignancies [4,5,6].

Integrating periodontal assessment into general healthcare and addressing both biological and social determinants is essential for comprehensive patient management. Numerous systemic diseases present with initial manifestations within the oral cavity, granting both dental and medical professionals a critical diagnostic role. These conditions span autoimmune, hematological, endocrine, dermatological, skeletal, and neoplastic disorders [7,8]. Oral manifestations are particularly prevalent in chronic inflammatory, autoimmune, and endocrine diseases. Disorders such as diabetes, anemia, celiac disease, and Crohn’s disease frequently involve the oral cavity. For instance, diabetes is associated with an increased risk of periodontitis and impaired oral wound healing [4]. Sjögren’s syndrome causes xerostomia, while anemia can present with oral pallor and atrophic tongue changes. Additionally, ulcers, glossitis, dry mouth, and burning sensations may indicate underlying conditions such as celiac disease, Crohn’s disease, or ulcerative colitis. Timely recognition of these oral signs can facilitate early diagnosis and improved management of systemic illnesses [4,9,10].

Despite the well-established connection between oral and systemic health, many medical and dental professionals receive insufficient training on this interrelationship. Oral health is often unrepresented in medical education, while dental curricula may inadequately address the systemic manifestation of disease. This educational gap contributed to missed diagnostic opportunities and delays in appropriate treatment [11,12]. Although the oral cavity is easily accessible for clinical examination, routine oral assessments remain underutilized in practice. Factors contributing to this include limited professional education, time constraints, and the absence of standardized protocols for interprofessional collaboration [13,14]. Promoting the awareness and integration of oral–systemic health requires multifaceted efforts, including public health campaigns, the enhancement of medical and dental education, the development of detailed clinical guidelines, and the implementation of educational initiatives, such as interdisciplinary workshops [15].

Interest in the relationship between oral and systemic health emerged in the 1980s, particularly with studies on periodontitis’ systemic effects [16], followed by a surge in research over the next two decades [17,18,19,20]. Current discourse emphasizes the systemic influence of the oral microbiome, the necessity of interdisciplinary care, and the importance of public education regarding the oral–systemic health connection [5,20,21]. While many professionals acknowledge the association between oral health and prevalent systemic conditions like diabetes and cardiovascular conditions, studies indicate a persistent gap in translating this knowledge into clinical practice. Furthermore, professionals often lack awareness of less commonly recognized associations, underscoring the need for continued education and practical integration of oral–systemic concepts in everyday healthcare delivery [22,23,24,25,26,27,28,29].

In Croatia, although the number of studies is limited, research has focused on the relationship between periodontal disease and systemic conditions, including liver, lung, and kidney disorders, as well as metabolic disturbances [27,28,29,30,31,32]. Research in transplant patients found that severe periodontitis is more common among those with compromised general health, particularly kidney disease, and is influenced by nutritional status, oral hygiene, and adherence to the Mediterranean diet [29,30]. Other studies found that obstructive sleep apnea correlates with altered salivary parameters and increased periodontal inflammation [31], while individuals with obesity show more dental tissue loss and poorer oral hygiene [32]. These findings highlight the importance of periodontitis in vulnerable groups and the need for integrated approaches in diagnosis, prevention, and care.

This study aimed to evaluate the knowledge and attitudes of dentists and physicians in Croatia regarding the relationship between oral and general health. It further explored their awareness of oral manifestations of systemic diseases, the connection between periodontitis and systemic conditions, and their self-assessed competence in managing these health issues. This research holds particular relevance in the context of the Croatian healthcare and educational systems, as it underscores the urgent need to strengthen interdisciplinary collaboration and to integrate oral health education more thoroughly into medical curricula in order to enhance patient care outcomes. The primary hypothesis was that physicians possess limited knowledge concerning the relationship between oral health and overall systemic health.

## 2. Materials and Methods

### 2.1. Study Design and Data Collection

This cross-sectional study was conducted at the Department of Restorative Dental Medicine and Endodontics, School of Medicine, University of Split, as part of a diploma thesis between 1 September and 30 October 2024. The study was approved by the Ethics Committee of the School of Medicine, University of Split (Class: 029-01/24-02/0001; Reg. No.: 2181-198-03-04-23-0028) and conducted in accordance with legal regulations and the Declaration of Helsinki. The methodology and results were reported in line with the Checklist for Reporting Results of Internet E-Surveys (CHERRIES) [33].

Participants were recruited using a combination of convenience and snowball sampling methods. An anonymous, structured, self-administered online questionnaire (Google Forms) with closed-ended questions was distributed via email to health centers across Croatia. Recipients were invited to complete the survey and encouraged to forward it to their colleagues. Participation was voluntary, with informed consent obtained at the beginning of the survey, which included details about the study purpose, inclusion criteria, number of questions, and expected duration. No personal identifiers were collected apart from education level, years of professional experience, gender, and workplace setting. Two reminders were sent during the data collection period. Although the survey was anonymous, measures were taken to minimize the risk of duplicate responses. The online survey platform was configured to accept only one response per device by using browser cookies. Additionally, participants were informed that they should complete the survey only once.

The survey was distributed to approximately 3000 physicians and dentists working in primary healthcare settings across Croatia. The inclusion criteria encompassed physicians and dentists who were currently practicing in Croatian primary care, with a minimum of one year of professional experience, proficient in the Croatian language, in possession of an active email address, and willing and able to complete the questionnaire. The exclusion criteria included incomplete or invalid responses, failure to meet inclusion criteria, retirement, or employment outside of Croatia.

According to national data, there are 16,107 physicians and 4013 dentists employed in Croatia [34]. The required sample size for adequate statistical power (n = 377) was calculated based on a total population of 20,120 doctors, with a 95% confidence level and a 5% margin of error, using Raosoft Sample Size Calculator software (Inc., RaoSoft^®^, Seattle, WA, USA) [35].

### 2.2. The Survey

A comprehensive literature review guided the development of the questionnaire, drawing on several previously published studies [6,14,20,36,37,38,39,40]. The survey was initially designed by a specialist in endodontics and restorative dental medicine in collaboration with a sixth-year dental student. To ensure content validity, the instrument was subsequently reviewed by a specialist in oral medicine. A pilot version was distributed to 40 healthcare professionals (20 physicians and 20 dentists) to evaluate the technical usability and clarity of the items. These participants were excluded from the final sample. Based on their feedback, minor modifications were made to improve question clarity.

The final questionnaire consisted of six sections, containing 55 closed-ended questions and two open-ended items related to age and years of professional experience. The average completion time was approximately 10 min. The first section focused on demographic and professional characteristics such as age, gender, education background, workplace setting, and professional experience. The second and third sections assessed knowledge of the association between periodontitis and systemic diseases (15 items) and knowledge of oral manifestations of systemic diseases (16 items), respectively. Responses were structured as “Yes”, “No”, or “I do not know” options, with “Yes” representing the correct answer. Each correct response was awarded one point, with the total scores categorized according to Bloom’s taxonomy as good (80–100%), moderate (60–79%), or poor (<60%) [41].

The fourth section examined participants’ knowledge acquired through formal education, as well as their attitudes toward oral health and its systemic connections. It also assessed their clinical practices and competencies related to the examination, management, and referral of patients presenting with oral lesions. Additionally, this section addressed perceived barriers to optimal care, such as insufficient knowledge, limited clinical resources, or lack of institutional support. The fifth section evaluated self-perceived knowledge, the frequency of encounters with patient with relevant conditions, and confidence in diagnosing and treating patients with oral lesions, manifestations of systemic diseases, and periodontitis. These responses were rated using a 5-point Likert scale and later grouped into three categories for analysis: limited (1–2), basic (3), and adequate (4–5) knowledge; rare (1–2), sometimes (3), and often (4–5) frequency; and unsure (1–2), neutral (3), and sure (4–5) levels of confidence.

### 2.3. Data Analysis

The data were analyzed using the Statistical Package for the Social Sciences, version 26.0 (SPSS, IBM Corp., Armonk, NY, USA), with the level of statistical significance set at *p* < 0.05. The primary outcomes of this study were the knowledge of physicians and dentists regarding the association between periodontitis and systemic diseases, as well as their knowledge about oral manifestations of systemic diseases. Categorical variables were expressed as numbers and percentages, while continuous variables were presented as mean and standard deviation. The normality of distribution for continuous variables was tested using the Shapiro–Wilk test. The Chi-square test and Fisher’s exact test were used to compare categorical variables between physicians and dentists. Differences in participants’ knowledge according to their sociodemographic and professional characteristics were assessed using a generalized linear regression model, with results presented as regression (β) coefficients and corresponding 95% confidence intervals (95% CI).

## 3. Results

Table 1 presents the demographic and professional characteristics of the respondents. A total of 529 participants took part in the study, including 238 (45.0%) dentists and 291 (55.0%) physicians. The average age of the participants was 40.1 ± 11.5 years (min = 24, max = 67) and the average work experience was 13.7 ± 10.8 years (min = 1, max = 42). Physicians had an average age of 41.5 ± 12.3 years (min = 24, max = 67) and an average work experience of 14.6 ± 11.4 years (min = 1, max = 42). Dentists had an average age of 38.5 ± 10.3 years (min = 24, max = 66) and an average work experience of 12.6 ± 10.0 years (min = 1, max = 40). The study included fourfold more women (N = 430, 81.1%) than men (N = 99, 18.9%).

Table 2 shows the distribution of correct answers to statements assessing knowledge about the association between periodontitis and systemic diseases. The average knowledge score of all participants was 6.8 ± 3.6 (min = 0, max = 15). The average score for physicians was 6.7 ± 3.8 (min = 0, max = 15), and for dentists, it was 7.0 ± 3.3 (min = 0, max = 15). Participants demonstrated the best knowledge on questions regarding the association of periodontitis with diabetes (N = 437, 82.6%), endocarditis (N = 369, 69.8%), and chronic gastritis/peptic ulcers (N = 361, 68.2%).

Table 3 presents the distribution of correct answers to statements assessing knowledge about oral manifestations of systemic diseases. The average knowledge score for all respondents was 10.0 ± 3.8 (min = 0, max = 16). The average score for physicians was 9.8 ± 3.8 (min = 1, max = 16), while for dentists it was 10.3 ± 3.8 (min = 0, max = 16). Respondents showed the best knowledge on questions regarding oral manifestations of Sjögren’s syndrome (N = 498, 94.1%) and anemia (N = 456, 86.2%).

Table 4 presents the responses to questions regarding the participants’ attitudes toward their own education, knowledge, diagnosis, and treatment of oral lesions. Most dentists stated that they had acquired knowledge about oral health (N = 211, 88.7%), whereas only a smaller proportion of physicians agreed with this statement (N = 82, 28.2%). A higher number of dentists reported regularly performing oral mucosa examinations (N = 213, 89.5%) compared to physicians (N = 125, 43.0%). Most of both dentists (N = 208, 87.4%) and physicians (N = 214, 73.5%) do not treat patients with oral lesions independently but refer them to a specialist. The most cited reason for not treating patients with oral lesions was a lack of sufficient experience and knowledge (N = 279, 52.7%).

Table 5 presents respondents’ answers regarding their self-assessment of knowledge, frequency of encountering, and confidence in diagnosing and treating patients with oral lesions, oral manifestations of systemic diseases, and periodontitis. Most dentists consider that they have adequate knowledge of oral diseases (N = 131, 55.0%), while most physicians believe they have basic knowledge (N = 158, 54.3%). Similar results were observed regarding their knowledge of periodontitis and oral manifestations of systemic diseases. In terms of confidence and treatment, dentists demonstrated greater confidence in diagnosing and treating patients with oral lesions, periodontitis, and oral manifestations of systemic diseases compared to physicians.

Table 6 presents the demographic and professional characteristics of the respondents in relation to the combined knowledge of oral manifestations of systemic diseases and the association between periodontitis and systemic conditions. Although dentists demonstrated better knowledge of oral manifestations of systemic diseases and connection between periodontitis and systemic conditions compared to physicians, this difference was not statistically significant. No statistically significant associations were found between the respondents’ demographic and professional characteristics and their combined knowledge of oral manifestations of systemic diseases and the relationship between periodontitis and systemic conditions.

## 4. Discussion

This study aimed to assess the knowledge and attitudes of physicians and dentists working in primary healthcare regarding the oral manifestations of systemic diseases and the association between periodontitis and systemic conditions. Overall, respondents demonstrated a low level of knowledge concerning the link between periodontitis and systemic diseases, whereases their knowledge of oral manifestations of systemic diseases was moderate. Although dentists achieved higher knowledge scores than physicians in both areas, these differences were not statistically significant. Therefore, the null hypothesis was confirmed, indicating that the overall knowledge level among participants was unsatisfactory. In contrast to these findings, previous studies have reported higher levels of knowledge among dental students and professionals [23,42]. For example, a study in Jordan found that medical students had significantly less knowledge about oral manifestations of systemic diseases than dental students [42]. Similarly, a Serbian study reported greater awareness among dental professionals regarding the relationship between periodontitis and systemic diseases, which differs from the results of this study [23].

The relationship between participants’ demographic and professional characteristics and their knowledge of oral manifestations of systemic diseases and the association between periodontal disease and systemic conditions was also explored. Although no statistically significant associations were identified, several notable trends emerged, offering valuable directions for future research. Higher knowledge levels were observed among female participants, those with postgraduate education, younger individuals, and those with less work experience. While these differences did not reach statistical significance, they are consistent with trends reported in previous studies. For example, a study from Serbia found that female medical and dental practitioners exhibited greater awareness of the relationship between periodontitis and systemic diseases, while years of experience and type of practice showed no significant impact on knowledge levels [23]. Similarly, a Jordanian study involving medical and dental students reported higher knowledge scores among female participants [42].

Physicians and dentists in this study demonstrated limited awareness of the association between periodontitis and systemic diseases, a finding consistent with research conducted in Turkey and Serbia [12,23]. The highest level of knowledge was related to the link between diabetes and periodontitis, followed by associations between endocarditis and peptic ulcers. In contrast, participants showed the lowest awareness regarding respiratory conditions—particularly pneumonia and chronic obstructive pulmonary disease (COPD)—as well as cognitive impairment. Comparable trends were observed in studies from India, Turkey, and Pakistan, where physicians reported strong knowledge of the diabetes–periodontitis connection, but limited awareness of links with neurological disorders, kidney diseases, osteoporosis, and obesity [43,44]. Awareness of the relationship between adverse pregnancy outcomes and periodontitis was also insufficient. Only 21.3% of physicians correctly identified this association, compared to 58.8% of dentists. These findings are in line with a study from Nigeria, where this link was the least recognized among physicians [45]. However, a study from Pakistan reported higher awareness among physicians [44], and similar results were found in India, where dental professionals outperformed gynecologists in knowledge of the impact of periodontitis on pregnancy outcomes [26]. While most participants across various studies recognized a general link between systemic health and periodontitis, more specific associations—particularly with cardiovascular, respiratory, or reproductive conditions—were less well understood. These findings support previous evidence indicating that healthcare professionals’ awareness of oral–systemic connections remains inadequate. For instance, a study from Portugal emphasized that, despite increasing scientific evidence linking oral health with chronic noncommunicable diseases, healthcare providers’ understanding remains limited [5]. Similarly, research from China noted that although clinical data increasingly highlight the role of periodontitis in systemic conditions such as diabetes and cardiovascular disease, this knowledge has yet to be fully integrated into routine clinical practice [4].

In this study, physicians and dentists demonstrated limited awareness of the oral manifestations of systemic diseases. These results are consistent with a study from France, where most dental practitioners showed limited knowledge about oral manifestations of systemic conditions [14]. The highest levels of knowledge in this study were recorded in questions regarding manifestations of Sjögren’s syndrome, systemic lupus erythematosus, and anemia. On the other hand, the lowest knowledge was observed concerning oral manifestations of neurodegenerative diseases. The smallest number of respondents recognized the connection between Parkinson’s disease and oral health. Besides Parkinson’s disease, participants showed poor knowledge about the oral manifestations of Alzheimer’s disease and multiple sclerosis. This highlights the need for additional education among healthcare professionals about neurodegenerative conditions and their impact on oral health. Furthermore, it is concerning that many respondents were unsure how to identify specific oral manifestations of diseases such as leukemia, multiple myeloma, gastrointestinal diseases, and others. This aligns with studies from the United States, which emphasize that oral manifestations are often the first sign of systemic diseases, but are frequently overlooked or misinterpreted [2,10]. Considering all of the above, the results of this research indicate the necessity of strengthening education and awareness among healthcare workers, especially physicians, about the importance of oral health and its implications for systemic health. The introduction of integrated modules on oral–systemic connections into medical and dental curricula, as well as the development of continuing education and clinical practice guidelines, is recommended. A study from the US investigated ethical challenges related to the integration of oral and general health. The authors emphasize that the fragmentation of healthcare harms patients, particularly regarding chronic diseases linked to oral health. The need for interprofessional collaboration and ethical responsibility among healthcare providers is highlighted. The study calls for policy and educational changes to enable the better integration of medicine and dentistry [13].

The results of this study revealed a significant disparity in attitudes between physicians and dentists regarding the education they received on oral health during their academic training. Specifically, 68.7% of dentists reported having received adequate education on oral health, in contrast to only 28.2% of physicians. Furthermore, almost all dental practitioners (99.8%) agreed that oral mucosal examination is an integral component of physical examination, whereas only 80% of physicians concurred with this statement. In terms of clinical behavior, 89.5% of dentists reported routinely performing oral cavity examinations in daily practice, compared to just 43% of physicians, highlighting a substantial gap in implementation between the two professional groups. A study from Pakistan showed that although dental practitioners were aware of the connection between oral and general health, their knowledge did not consistently translate into practice [21]. These findings align with those of the present study. Referral practices for patients with oral diseases differed between physicians and dentists in this research. Dentists most frequently referred patients to specialists in oral medicine and periodontology, whereas physicians more often referred patients to general dental practitioners or maxillofacial surgeons. This indicates a lack of awareness among physicians about the role of oral medicine specialists and periodontologists in treating oral diseases. Interestingly, although most respondents recognized the existence of a link between oral and general health, self-assessed knowledge among physicians revealed uncertainty and doubt regarding their competence in identifying oral manifestations of systemic diseases. The most common reasons given for not treating these patients were insufficient experience and knowledge, followed by lack of confidence and time constraints. A study from the United States investigating dental practitioners’ attitudes toward oral–systemic connections concluded that most participants acknowledged this relationship, but only a small number regularly applied this knowledge in practice, which corresponds with the current study. The lack of education and clinical collaboration was identified as the main cause [20]. This points to a gap between the perceived importance and actual clinical practice, which is further supported by research from India and Saudi Arabia showing that insufficient knowledge among physicians often leads to poor collaboration with dental practitioners [6,11].

A self-assessment was conducted among physicians and dentists to evaluate their perceived knowledge and confidence in diagnosing and managing oral lesions, oral manifestations of systemic diseases, and periodontal conditions. When asked about their knowledge of oral lesions, 55% of dentists reported having adequate knowledge, compared to only 27.8% of physicians. This discrepancy reflects greater confidence among dental professionals in their ability to understand and recognize oral lesions. Moreover, statistically significant differences were identified in confidence levels related to the diagnosis and treatment of oral lesions, consistently favoring dentists over physicians. In the area of oral manifestations of systemic diseases, 36% of dentists considered their knowledge adequate, whereas only 17% of physicians reported the same. The most pronounced disparity appeared in relation to periodontitis: 85% of dentists rated their knowledge as adequate, compared to just 10% of physicians. Moreover, 42% of physicians assessed their knowledge as limited, while only 1.7% of dentists did so. These findings clearly point to greater self-perceived competence among dental professionals in the recognition and management of periodontitis. Similar results were reported in a UK-based study, where only 35% of physicians expressed confidence in diagnosing and managing oral conditions [46].

Several limitations should be acknowledged. Data collection was based on an online survey that relied on participants’ self-assessment, introducing the potential for subjective bias. Nonresponse bias is also possible, as those with limited knowledge might have opted not to participate, thereby affecting the representativeness of the sample. The relatively small and gender-imbalanced sample—predominantly female—further limits generalizability. Additionally, all knowledge questions had “Yes” as the expected correct answer, which may have led to an overestimation of knowledge levels. The average survey completion time of 10 min could have resulted in incomplete submissions or early dropouts. A high proportion of young and female participants may have introduced further bias, possibly distorting the results compared to a more demographically balanced population. The exclusive use of closed-ended questions may have limited the depth of response and failed to fully capture the participants’ attitudes. Conducting the survey online also removed opportunities for observational validation, preventing direct assessment of clinical behavior and actual competencies.

Despite these limitations, the findings offer valuable insights into the existing knowledge gaps and confidence levels among primary healthcare professionals regarding oral–systemic health connections. Recognizing these gaps enables the development of targeted educational strategies aimed at improving both clinical awareness and competence. The inclusion of primary care professionals enhances the practical relevance of the results, reflecting real-world challenges in day-to-day patient management. These outcomes may guide future curriculum development in both medical and dental education and support broader efforts toward interdisciplinary healthcare integration. Moving forward, emphasis should be placed on strengthening collaboration between physicians and dentists and embedding oral health within a holistic model of patient care, ultimately contributing to more effective and comprehensive healthcare delivery. Further research involving a larger and more diverse sample of healthcare providers across Croatia is strongly recommended. Objective knowledge assessments should be incorporated to minimize subjective bias. Additionally, longitudinal and qualitative methods—such as focus groups—could provide a deeper understanding of barriers to applying knowledge in clinical settings and help to evaluate the success of interprofessional models. The integration of oral health topics into medical curricula and the provision of continuous professional development are essential steps toward improving patient care, aligning with the World Health Organization’s recommendations on the integration of oral and general health [42,47].

## 5. Conclusions

Physicians and dentists in Croatia demonstrated limited knowledge regarding the connection between periodontitis and systemic diseases, along with a moderate understanding of the oral manifestations of systemic conditions. While dentists generally scored slightly higher, the difference was not statistically significant. The highest levels of awareness were observed for conditions such as diabetes, infective endocarditis, peptic ulcer disease, Sjögren’s syndrome, systemic lupus erythematosus, and anemia. Dentists reported feeling better educated during their formal training about the link between oral and general health compared to physicians. However, both professional groups lacked confidence in diagnosing and managing patients with periodontitis, oral lesions, and systemic disease-related oral manifestations. Despite this, they expressed a strong willingness to pursue further education on the topic. These findings underscore the urgent need to enhance interdisciplinary education and training to improve patient care outcomes. In particular, continuing education programs should incorporate targeted modules focusing on the oral–systemic health connection, practical diagnostic skills, and collaborative management strategies, in order to strengthen healthcare professionals’ competence and interprofessional coordination in this critical area.

## Figures and Tables

**Table 1:** Demographic and professional characteristics of respondents.

Characteristic		Total N = 529	DMD N = 238	MD N = 291	*p*-Value
Gender	Man	99 (18.7)	48 (20.2)	51 (17.5)	0.253
	Woman	430 (81.1)	190 (79.8)	240 (82.5)	
Age	≤30	158 (29.8)	74 (31.1)	84 (28.9)	0.018 *
	31–40	142 (26.8)	76 (31.9)	66 (22.7)	
	41–50	115 (21.7)	49 (20.6)	66 (22.7)	
	≥51	114 (21.6)	39 (16.4)	75 (25.8)	
Education	MD/DMD	486 (91.9)	216 (90.8)	270 (92.8)	0.245
	MSc/PhD	43 (8.1)	22 (9.2)	21 (7.2)	
Professional experience	≤5	176 (33.3)	83 (34.9)	93 (32.0)	0.032
	6–10	79 (14.9)	46 (19.3)	33 (11.3)	
	11–20	138 (26.1)	59 (24.8)	79 (27.1)	
	≥20	136 (25.7)	50 (21.0)	86 (29.6)	
Workplace setting	Private office	51 (9.6)	63 (26.5)	41 (14.1)	≤0.001 *
	Health center	478 (90.4)	175 (73.5)	250 (85.9)	

Abbreviations: DMD, Doctor of Dental Medicine; MD, Doctor of Medicine. Data are presented as frequencies (percentages). Chi-square test or Fisher’ exact test, * *p* < 0.05.

**Table 2:** Distribution of correct answers (‘’Yes’’) to statements assessing participants’ knowledge about the association between periodontitis and systemic diseases.

Question	Total N = 529	DMD N = 238	MD N = 291	*p*-Value
Endocarditis (“Yes”)	369 (69.8)	183 (76.9)	186 (63.9)	≤0.001 *
Diabetes mellitus (“Yes”)	437 (82.6)	217 (91.2)	220 (75.6)	≤0.001 *
Obesity (“Yes”)	238 (45.0)	99 (41.6)	139 (47.8)	0.161
Metabolic syndrome (“Yes”)	325 (61.3)	144 (60.5)	181 (62.2)	0.720
Atherosclerosis (“Yes”)	193 (36.5)	91 (38.2)	102 (35.1)	0.469
Chronic kidney disease (“Yes”)	201 (38.0)	86 (36.1)	115 (39.5)	0.471
Recurrent pneumonia (“Yes”)	137 (25.9)	45 (18.9)	92 (31.6)	≤0.001 *
COPB (“Yes”)	160 (30.2)	63 (26.5)	97 (33.3)	0.106
Chronic gastritis/peptic ulcer (“Yes”)	361 (68.2)	136 (57.1)	225 (77.3)	≤0.001 *
Rheumatoid arthritis (“Yes”)	244 (46.1)	94 (39.5)	150 (51.5)	0.007 *
Head and neck tumors (“Yes”)	226 (42.7)	85 (35.7)	141 (48.5)	0.004 *
Adverse pregnancy outcomes (“Yes”)	202 (38.2)	140 (58.8)	62 (21.3)	≤0.001 *
Cognitive deficit and dementia (“Yes”)	163 (30.8)	82 (34.5)	81 (27.8)	0.108
Alzheimer’s disease (“Yes”)	170 (32.1)	86 (36.1)	84 (28.9)	0.076
Osteoporosis (“Yes”)	223 (42.2)	125 (52.5)	98 (33.7)	≤0.001 *

Abbreviations: DMD, Doctor of Dental Medicine; MD, Doctor of Medicine. Data are presented as frequencies (percentages). Chi-square test or Fisher’s exact test, * *p* < 0.05.

**Table 3:** Distribution of correct answers (“Yes”) to statements assessing participants’ knowledge of oral manifestations of systemic diseases.

Question	Total N = 529	DMD N = 238	MD N = 291	*p*-Value
Sjögren’s syndrome (“Yes”)	498 (94.1)	223 (93.7)	275 (94.5)	0.713
Systemic lupus erythematosus (“Yes”)	452 (85.4)	202 (84.9)	250 (85.9)	0.804
Systemic sclerosis (“Yes”)	411 (77.2)	163 (68.5)	248 (85.2)	≤0.001 *
Lichen planus (“Yes”)	400 (75.6)	218 (91.6)	182 (62.5)	≤0.001 *
Bullous diseases (“Yes”)	318 (60.1)	186 (78.2)	132 (45.4)	≤0.001 *
Ulcerative colitis/Crohn’s disease (“Yes”)	401 (75.8)	171 (71.8)	230 (79.0)	0.066
Celiac disease (“Yes”)	309 (58.4)	125 (52.5)	184 (63.2)	0.013 *
Anemia (“Yes”)	456 (86.2)	198 (83.2)	258 (88.7)	0.077
Thrombocytopenia (“Yes”)	383 (72.4)	180 (75.6)	203 (69.8)	0.143
Diabetes mellitus (“Yes”)	433 (81.9)	218 (91.6)	215 (73.9)	≤0.001 *
Addison’s disease (“Yes”)	185 (35.0)	96 (40.3)	89 (30.6)	0.022 *
Multiple sclerosis (“Yes”)	143 (27.0)	64 (26.9)	79 (27.1)	1.000
Alzheimer’s disease (“Yes”)	149 (28.2)	64 (26.9)	85 (29.2)	0.562
Parkinson’s disease (“Yes”)	138 (26.1)	67 (28.2)	71 (24.4)	0.371
Leukemia (“Yes”)	372 (70.3)	172 (72.3)	200 (68.7)	0.391
Multiple myeloma (“Yes”)	277 (52.4)	123 (51.7)	154 (52.9)	0.793

Abbreviations: DMD, Doctor of Dental Medicine; MD, Doctor of Medicine. Data are presented as frequencies (percentages). Chi-square test or Fisher’s exact test, * *p* < 0.05.

**Table 4:** Participants’ attitudes on education, knowledge, diagnosis, and treatment of oral lesions.

Characteristic		Total N = 529	DMD N = 238	MD N = 291	*p*-Value
Acquired knowledge during studies	Yes	293 (55.4)	211 (88.7)	82 (28.2)	≤0.001 *
Interest in further education	Yes	478 (90.4)	220 (92.4)	258 (88.7)	0.182
Considering the relationship between oral and general health	Yes	527 (99.6)	237 (99.6)	290 (99.7)	1.000
Considering the examination of the oral mucosa as an integral part of the physical examination	Yes	472 (89.2)	237 (99.6)	235 (80.8)	≤0.001*
Practicing oral mucosa examination	Yes	338 (63.9)	213 (89.5)	125 (43.0)	≤0.001 *
Treatment of oral mucosa lesions	Independently	107 (20.2)	30 (12.6)	77 (26.5)	≤0.001 *
	Specialist referral	422 (79.6)	208 (87.4)	214 (73.5)	
Referral of a patient with oral lesions to a specialist (multiple answers possible)	Independently	109 (20.6)	46 (19.3)	63 (21.6)	0.519
	MD	29 (5.5)	17 (7.1)	12 (4.1)	0.178
	DMD	223 (42.2)	9 (3.8)	214 (73.5)	≤0.001 *
	Dermatologist	36 (6.8)	17 (7.1)	19 (6.5)	0.863
	Maxillofacial surgeon	190 (35.9)	61 (25.6)	129 (44.3)	≤0.001 *
	Oral surgeon	220 (41.6)	101 (42.4)	119 (40.9)	0.724
	Oral medicine specialist	341 (64.5)	227 (95.4)	114 (39.2)	≤0.001 *
	Periodontologist	165 (31.2)	110 (46.2)	55 (18.9)	≤0.001 *
	Other specialists	70 (13.2)	20 (8.4)	55 (18.9)	0.003 *
Reasons for not treating patients (multiple responses possible)	Insufficient financial compensation	13 (2.5)	8 (3.4)	5 (1.7)	0.266
	Insufficient experience or knowledge	279 (52.7)	110 (46.2)	169 (58.1)	0.007 *
	Lack of confidence	185 (35.0)	43 (18.1)	142 (48.8)	≤0.001 *
	Lack of time	95 (18.0)	41 (17.2)	54 (18.6)	0.733
	Other	182 (34.4)	116 (48.7)	66 (22.7)	≤0.001 *

Abbreviations: DMD, Doctor of Dental Medicine; MD, Doctor of Medicine. Data are presented as frequencies (percentages). Chi-square test or Fisher’s exact test, * *p* < 0.05.

**Table 5:** Self-assessment of knowledge, frequency of patient encounters, and confidence in diagnosing and treating patients with oral lesions, oral manifestations of systemic diseases, and periodontitis.

Characteristic		Total N = 529	DMD N = 238	MD N = 291	*p*-Value
Self-assessed knowledge of oral diseases	Limited	72 (13.6)	20 (8.4)	51 (17.9)	≤0.001 *
	Basic	245 (46.3)	87 (36.6)	158 (54.3)	
	Adequate	212 (40.1)	131 (55.0)	81 (27.8)	
Frequency of patient visits with oral diseases	Rarely	142 (24.0)	64 (26.9)	78 (26.8)	0.496
	Sometimes	274 (51.8)	122 (51.3)	152 (52.2)	
	Often	113 (21.4)	52 (21.8)	61 (20.9)	
Confidence in diagnosing patients with oral diseases	Unconfident	80 (15.1)	17 (7.1)	63 (21.6)	≤0.001 *
	Neutral	168 (31.8)	71 (29.8)	97 (33.3)	
	Confident	281 (53.1)	150 (63.0)	131 (45.0)	
Confidence in treating patients with oral diseases	Unconfident	98 (18.5)	33 (13.9)	65 (38.8)	≤0.001 *
	Neutral	200 (37.8)	80 (33.6)	130 (44.7)	
	Confident	231 (43.7)	125 (52.5)	48 (16.5)	
Self-assessed knowledge of oral manifestations of systemic diseases	Limited	117 (22.1)	46 (19.3)	71 (24.4)	≤0.001 *
	Basic	275 (52.0)	105 (44.1)	170 (58.4)	
	Adequate	137 (25.9)	87 (36.5)	50 (17.1)	
Frequency of patient visits with oral manifestations of systemic diseases	Rarely	182 (34.4)	72 (30.3)	110 (37.8)	≤0.001 *
	Sometimes	265 (50.1)	116 (48.7)	149 (51.2)	
	Often	82 (15.5)	50 (21.0)	32 (11.0)	
Confidence in diagnosing patients with oral manifestations of systemic diseases	Unconfident	164 (31.0)	54 (22.7)	110 (37.8)	≤0.001 *
	Neutral	225 (42.5)	101 (42.4)	124 (42.6)	
	Confident	140 (26.5)	83 (34.9)	57 (19.6)	
Confidence in treating patients with oral manifestations of systemic diseases	Unconfident	179 (33.8)	66 (27.7)	113 (38.8)	≤0.001 *
	Neutral	237 (44.8)	107 (45.0)	130 (44.7)	
	Confident	113 (21.3)	65 (27.3)	48 (16.5)	
Self-assessed knowledge of periodontal diseases	Limited	127 (24.0)	4 (1.7)	123 (42.3)	≤0.001 *
	Basic	169 (31.9)	31 (13.0)	138 (47.4)	
	Adequate	233 (44.1)	203 (85.3)	30 (10.3)	
Frequency of patient visits with periodontal diseases	Rarely	122 (23.1)	1 (0.4)	121 (41.6)	≤0.001 *
	Sometimes	123 (23.3)	19 (8.0)	104 (35.7)	
	Often	284 (53.7)	218 (91.6)	66 (22.6)	
Confidence in diagnosing patients with periodontal diseases	Unconfident	112 (21.2)	2 (0.8)	110 (37.8)	≤0.001 *
	Neutral	110 (20.8)	8 (3.4)	102 (35.1)	
	Confident	307 (58.0)	228 (95.8)	78 (27.1)	
Confidence in treating patients with periodontal diseases	Unconfident	154 (29.1)	5 (2.1)	149 (51.2)	≤0.001 *
	Neutral	125 (23.6)	17 (7.1)	108 (37.1)	
	Confident	250 (47.3)	216 (90.8)	34 (11.7)	

Abbreviations: DMD, Doctor of Dental Medicine; MD, Doctor of Medicine. Data are presented as frequencies (percentages). Chi-square test or Fisher’s exact test, * *p* < 0.05.

**Table 6:** Demographic and professional characteristics of respondents concerning the combined knowledge of oral manifestations of systemic diseases and the association between periodontitis and systemic conditions.

Characteristic		Periodontitis and Systemic Diseases β (95% CI)	*p*-Value	Oral Manifestations β (95% CI)	*p*-Value
Gender	Man	Reference		Reference	
	Woman	0.038 (−0.763–0.839)	0.925	−0.048 (−0.890–0.794)	0.911
Age	≤30	Reference		Reference	
	31–40	−0.366 (−2.048–1.316)	0.670	−0.041 (−1.809–1.728)	0.964
	41–50	−0.796 (−2.737–1.146)	0.422	−0.382 (−2.423–1.660)	0.714
	≥51	−0.744 (−2.995–1.508)	0.517	−0.516 (−2.883–1.851)	0.669
Education	MD/DMD	Reference		Reference	
	MSc/PhD	0.236 (−0.937–1.409)	0.694	−1.027 (−2.260–0.207)	0.103
Profession	DMD	Reference		Reference	
	MD	−0.318 (−0.959–0.322)	0.330	−0.520 (−1.193–0.153)	0.130
Professional experience	≤5	Reference		Reference	
	6–10	1.065 (−0.694–2.825)	0.235	−0.623 (−2.473–1.227)	0.509
	11–20	0.645 (−1.149–2.439)	0.481	−0.414 (−2.300–1.473)	0.667
	≥20	1.393 (−0.766–3.553)	0.206	−0.076 (−2.346–2.195)	0.948
Workplace setting	Private practice	Reference		Reference	
	Health center	0.513 (−0.321–1.346)	0.228	−0.383 (−1.260–0.493)	0.391

Data are presented as β (95% CI). The reference knowledge category is “low”. Abbreviations: β, β-regression coefficient, 95% CI, 95% confidence interval; DMD, Doctor of Dental Medicine; and MD, Doctor of Medicine.

## Data Availability

Data supporting the findings of this study are available on request.
